# [Retracted] 14‑3‑3β exerts glioma‑promoting effects and is associated with malignant progression and poor prognosis in patients with glioma

**DOI:** 10.3892/etm.2024.12368

**Published:** 2024-01-08

**Authors:** Liang Liu, Zhixiong Liu, Hao Wang, Long Chen, Fuqiang Ruan, Jihui Zhang, Yi Hu, Hengshan Luo, Shuai Wen

Exp Ther Med 15:2381–2387, 2015; DOI: 10.3892/etm.2017.5664

Following the publication of the above paper, it was drawn to the Editors’ attention by a concerned reader that certain of the colony-formation assay data shown in Fig. 1B, the cell-cycle assay data in Fig. 2A, the cell apoptotic assay data in Fig. 2B, the Transwell cell migration assay data in Fig. 3B and tumour images in Fig. 4A were strikingly similar to data appearing in different form in other articles written by different authors at different research institutes (some of which have been retracted), which had either already been published elsewhere prior to the submission of this paper to *Experimental and Therapeutic Medicine*, or were under consideration for publication at around the same time. Owing to the fact that certain of these data had already apparently been published previously, the Editor of *Experimental and Therapeutic Medicine* has decided that this paper should be retracted from the Journal. The authors were asked for an explanation to account for these concerns, but the Editorial Office did not receive a reply. The Editor apologizes to the readership for any inconvenience caused.

